# A Program on Prevention of Mother to Child Transmission of HIV at Government Hospital, Tiruchengode Taluk, Namakkal District

**DOI:** 10.4103/0970-0218.55298

**Published:** 2009-07

**Authors:** Srijayanth Parameshwari, Mini S Jacob, JJ Vijayakumari, Diana Shalini, Mary K Sushi, MR Sivakumar

**Affiliations:** Department of Experimental Medicine, The Tamilnadu Dr. MGR Medical University, Chennai, India; 1 Department of Namakkal PPTCT Center, Namakkal District Headquarter Hospital, Namakkal, India; 2 Department of Tiruchengode PPTCT Center, Tiruchengode Government Hospital, Namakkal, India

## Introduction

UNAIDS states that mother to child transmission (MTCT) is the largest source of HIV infection in children below the age of 15 years.(1) According to National AIDS Control Organization (NACO), it is estimated that about 30,000 infants acquire HIV infection each year.(2) Tamil Nadu Sentinel surveillance for 2004, showed that the median positivity rate of HIV infection among the antenatal women was 0.65% (range 0-3.7%)(3) and in 2005 it was 0.50%.

Prevention of mother to child transmission (PMTCT) program has been established at the Tiruchengode government hospital (Tiruchengode taluk, Namakkal district) in November 2002 by the Department of Experimental Medicine of the Tamilnadu Dr. MGR Medical University. This was the first PMTCT program in India that has been established at a taluk hospital. The objective of the program was to establish a PMTCT center and offer antiretroviral prophylaxis to all HIV positive pregnant women and their infants.

## Materials and Methods

Group counseling and education in Tamil (local language) on health and hygiene during pregnancy, importance of regular antenatal visits, nutrition, importance of HIV testing, HIV prevention and infant feeding issues were stressed to all pregnant women visiting the antenatal clinic. After obtaining written informed consent, HIV testing was performed followed with individual post test counseling. HIV seronegative women are counseled on HIV prevention and risk reduction behaviors. For HIV seropositive pregnant women, individual post test counseling included psychological support, ARV prophylaxis, infant feeding options, disclosure issues, couple-counseling sessions, postpartum follow up, nutrition, and prevention of pregnancy.

Blood was collected by finger-stick and rapid test was performed using CombAIDS. If the test was reactive, blood was collected by venipuncture and two other rapid tests were performed (EIA Comb and Tridot).

Tablet nevirapine (single dose of 200 mg at the onset of labor) was provided to pregnant women to take home in the 3^rd^ trimester. Nevirapine syrup (2 mg/kg body weight) was offered to the infants within 72 hours after birth. HIV DNA PCR was performed for the infants at six months of age and HIV rapid test was done at 18 months.

## Results

Over a period of five years, 7866 pregnant women accessed the PMTCT services. Group counseling was provided in batches of 2-8 women with an average of 3 women and the time taken was 20-40 minutes. Fifty six antenatal women (0.77%) tested positive [Table 1]. Forty seven of them (83%) received nevirapine. Nine did not receive nevirapine because three gave false addresses, two shifted out of the locality and one patient expired. Three antenatal women were yet to deliver.

**Table 1:** Results of sample tested for HIV in pregnant women at the Thiruchengode government hospital

Time period	ANC women counseled	Tested	HIV+ (%)
Oct-Dec 2002	438	438	5 (1.14)
Jan-Dec 2003	1639	1639	10 (0.61)
Jan-Dec 2004	1357	1357	14 (1.03)
Jan-Dec 2005	1881	1881	12 (0.64)
Jan- Dec 2006	1349	1349	13 (0.96)
Jan- Dec 2007	1640	1640	7 (0.42)
Total	7866	7866	56 (0.7)

All of the seropositive pregnant women were married and were below 30 years. None had any opportunistic infections (O.I.). 75% (35/47) had vaginal delivery while 12 (25%) underwent caesarian section.

Forty six infants were born and one was stillbirth. The mean birth weight of the infants was 2.6 kg. There were no congenital abnormalities. Two infants survived for a day and then died of pneumonitis and acute gastroenteritis, respectively. One infant was diagnosed with protein energy malnutrition at the age of 5 months and expired.

Forty four (44/46) infants received nevirapine syrup. Thirty two of them received nevirapine in the government hospital, 8 during the home visit and 4 infants received nevirapine in private hospital. Twenty infants who completed 6 months of age tested negative using HIV DNA PCR. HIV rapid test was performed for 14 of the infants at 18 months and two were found to be HIV positive.

## Discussion

The Department of Experimental Medicine of the Tamilnadu Dr. MGR Medical University initiated PMTCT program at the Government Hospital, Thiruchengode taluk, Namakkal District in Oct 2002. This program is unique that it offers services to those women who are able to visit the government hospital at taluk level.

Rouzioux *et al*. observed that perinatal transmission could occur during antepartum, intrapartum and after delivery through breast milk.(4) Therefore, strategies for PMTCT would involve HIV education, voluntary counseling and testing (VCT) for pregnant women and providing antiretroviral prophylaxis to them and their infants.

In this study, the acceptability of HIV education and VCT is 100%. The factors leading to high rates of acceptance for HIV testing are cultural; where the rural women regard the health care workers with high respect and feel obligated to get tested in spite of testing not being mandatory, Group dynamics may also play a role, since if one person gets tested, the rest in the group also get tested.

Rapid HIV testing is beneficial to pregnant women. The test is simple and easy to use at the rural settings. Moreover, the test results are provided to the pregnant women on the same day. In a study done among women in northern Thailand, Liu *et al.* observed that 56% of the women preferred blood collection by finger-stick and 79% of women favored rapid test method to venepuncture.(5)

Single-dose nevirapine prophylaxis to mother and infant is widely used in resource-constrained settings for PMTCT programs. The simplicity and low cost of nevirapine's single dose regimen suggest that this highly efficacious drug might be very useful in rural settings. As the tablet does not require refrigeration, it can be offered to the mother in the last week of trimester. In this study, 83% of the pregnant women received nevirapine prophylaxis while 96% (44/46) infants received nevirapine syrup.

HIV DNA PCR was performed at 6 months of age for the infants. Dunn *et al.* reported a meta-analysis of published data from 271 infected children indicated that HIV DNA PCR was sensitive for the diagnosis of HIV infection during the neonatal period. Thirty-eight percent (90% confidence interval [CI] = 29%-46%) of infected children had positive HIV DNA PCR tests by age 48 hours. No substantial change in sensitivity during the first week of life was observed, but sensitivity increased rapidly during the second week, with 93% of infected children (90% CI = 76-97%) testing positive by PCR by age 14 days. By age 28 days, HIV DNA PCR has 96% sensitivity and 99% specificity to identify HIV proviral DNA in peripheral blood mononuclear cells (PBMCs).(6)

In this study, 20 infants born to the seropositive women were HIV negative at 6 months of age when tested with in house qualitative HIV DNA PCR. WHO recommendation is that a diagnosis of HIV-1 infection can be made on the basis of two positive HIV-1 DNA or RNA assay results. However, in this study, 14 of the above infants were tested at 18 months of age and two (14%) tested positive by HIV rapid tests. Both these infants were male infants. Both the infants and their mothers received Nevirapine prophylaxis. One was born through vaginal delivery and the other was by caesarian section. One infant received exclusive breast-feeding for the first four months and the other has received mixed feeds. The diagnosis of HIV-1 infection among infants and young children with a history of breastfeeding is more difficult because of continuing exposure to the virus postnatally.(7) The HIV positive infants in this study may have been infected through breast feeding.

## Conclusions

PMTCT programs are feasible in government hospitals where resources are limited. Rural pregnant women are receptive to voluntary counseling and testing. Through this program, rural pregnant women were educated on HIV/AIDS and PMTCT. Simple intervention strategies for PMTCT will reduce the incidence of pediatric HIV infection in India.
